# Data collection of biomedical data and sensing information in smart rooms

**DOI:** 10.1016/j.dib.2023.108922

**Published:** 2023-01-20

**Authors:** Yuichi Sei, Akihiko Ohsuga

**Affiliations:** a The University of Electro-Communications, 1-5-1, Chofugaoka, Chofu, Tokyo 1828585, Japan; b JST, PRESTO, 4-1-8, Honcho, Kawaguchi, Saitama 3320012, Japan

**Keywords:** IoT, Smart rooms, Personal information, Sensing data, Activity data, Biomedical data, Sleep data

## Abstract

This paper presents a new dataset, including behavioral, biometric, and environmental data, obtained from 23 subjects each spending 1 week to 2 months in smart rooms in Tokyo, Japan. The approximate duration of the experiment is 2 years. This dataset includes personal data, such as the use of home appliances, heartbeat rate, sleep status, temperature, and illumination. Although there are many datasets that publish these data individually, datasets that publish them all at once, tied to individual pseudo IDs, are valuable. The number of days for which data were obtained was 488, the number of records was 18,418,359, and the total size of the obtained data was 2.76 GB. This dataset can be used for machine learning and analysis for tips on getting a good night's sleep, for example.

**Specifications Table**

**Table:** 

Subject:	Data Mining and Statistical Analysis
Specific subject area:	Analysis of Human Behavior
Type of data:	Table
How the data were acquired:	A system was built to collect data from 10 sensing systems. Sensing systems include MW-PAL-MAG-0, MW-B-PAL-P, TWE-LITE-R, and TWE-l-DI-W (MONO WIRELESS) for open/closed sensor data, WS-USB02-PIR for human detection data, MZK-EX300NM (PLANEX COMMUNICATIONS INC.) and Power Consumption Monitor (TEPCO Energy Partner, Incorporated) for electric power data, Joy-Con (Nintendo Co., Ltd.) for activity game log data, Fitbit Aria 2 (Fitbit, Inc.) for body composition data, Fitbit Charge3 (Fitbit, Inc.) for activity level, sleep, and heart rate data, Withings Sleep (Withings) for sleep data, and Nature Remo and WS-USB01-THP for environmental data.
Data format:	Raw
Description of data collection:	Two smart rooms were prepared in Tokyo, Japan, where more than 30 subjects lived individually for 1 week to 2 months, and data on their use of home appliances, biometric information, and activity status were collected. The experiment ran from March 2020 to April 2022. Due to privacy reasons, the dataset contains 23 subjects.
Data source location:	Institution: The University of Electro-Communications City/Town/Region: Chofu, Tokyo Country: Japan
Data accessibility:	Repository name: Mendeley Data Data identification number: 10.17632/ycy9zmhts5.1 Direct link to the dataset: https://dx.doi.org/10.17632/ycy9zmhts5.1

## Value of the Data

• This dataset was created by capturing people's daily behavioral and biometric information, as well as the room's environmental information. While there are many datasets that provide each piece of information individually, there are very few that provide all of this information in one place, tied to each person.
• It would be beneficial for organizations to provide machine learning applications that predict future behavior based on historical data.
• It can also perform machine learning tasks to predict biometric information, such as sleep status and pulse rate for the night, based on the day's activities.
• This dataset contains sensing data that have not been preprocessed, allowing the developer to perform any preprocessing.

## 1 Data Description

The collected data fall into three major categories: behavioral data, biometric data, and environmental data. Behavioral data include open/closed sensor data, human detection sensor data, electric power data, and activity game log data. Biometric data include body composition data, activity level data, sleep data, and heartrate data. Environmental data include humidity, and illuminance, for example. The detailed definition and statistical information of the data is shown in the following subsections. Because the dataset includes various types of data, considerable statistical analysis can be conducted. In this section, only the representative analysis of each data type is described.

### 1.1 Data Definition

#### 1.1.1 Open/Closed Sensor Data

**Table:** 

Column name	Column ID	Type	Explanation
ID	id	Int	ID of each datum
Room ID	room	Int	Room ID where the experiment took place
Sensor name	name	String	PAL
Date	date	DATETIME	Measurement time
Value	val	String	Open/Closed
Sequence	sequence_number	Int	Numbering by each sensor
Physical sensor ID	sid	String	Physical ID of the sensor
Logical sensor ID	logical_id	Int	Logical ID of the sensor
Place	place	String	Name of sensor location
Time stamp	time stamp	DATETIME	Time stored in database

**Fig. 1:**
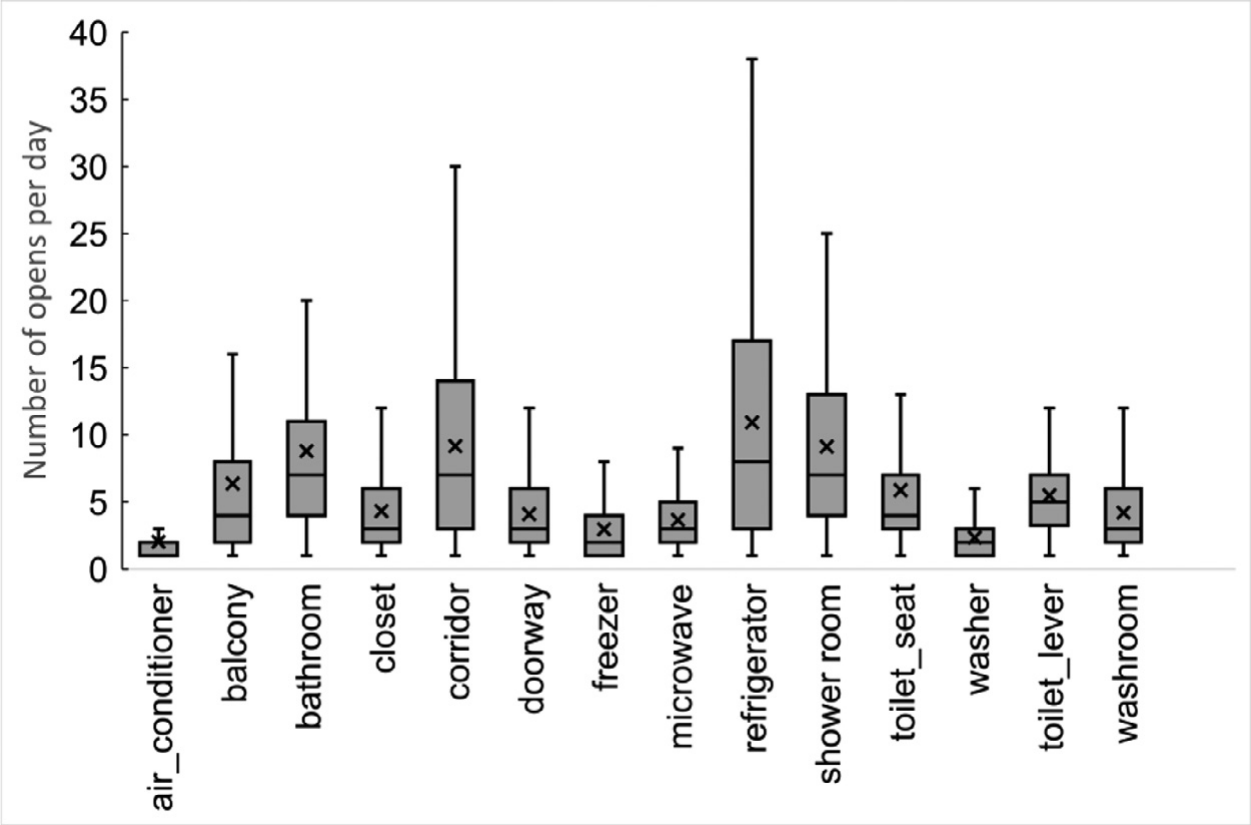
Number of opens per day based on the open/closed sensor data.

These data represent the time of opening and closing of appliances, corridors, and doors. The boxplot of the number of opens per day based on these data is shown in Fig. 1. For example, subjects used the refrigerator about 10 times on average per day.

#### 1.1.2 Human Detection Sensor Data

**Table:** 

Column name	Column ID	Type	Explanation
ID	id	Int	ID of each datum
Sensor name	name	String	PIR
Date	date	DATETIME	Measurement time
Room ID	room	Int	Room ID where the experiment took place
Value	val	Int	Detection sensitivity
Place	place	String	Location
Time stamp	time stamp	DATETIME	Time stored in database

**Fig. 2:**
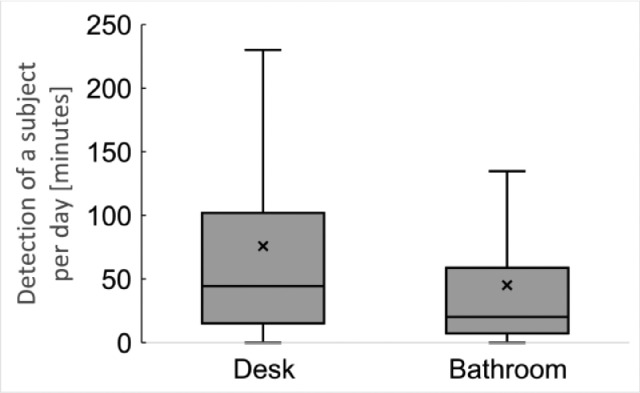
Detection of a subject per day based on the human detection sensor data.

These data represent the time of detecting human existence. The boxplot of the detection of a subject per day based on these data is shown in Fig. 2. The subjects existed in front of the desk about 75 min and in the bathroom about 45 min on average per day.

#### 1.1.3 Electric Power Data

##### 1.1.3.1 Electric Power Data by MZK-EX300NM

**Table:** 

Column name	Column ID	Type	Explanation
ID	id	Int	ID of each datum
Sensor name	name	String	MZK-EX300NM
Date	date	DATETIME	Measurement time
Room ID	room	Int	Room ID where the experiment took place
Place	place	String	Location
Electric power	watt	Float	Electric power (WATT)
Voltage	volt	Float	Voltage (VOLT)
Ampere	ampere	Float	Ampere (AMPERE)
Time stamp	time stamp	DATETIME	Time stored in database

**Fig. 3:**
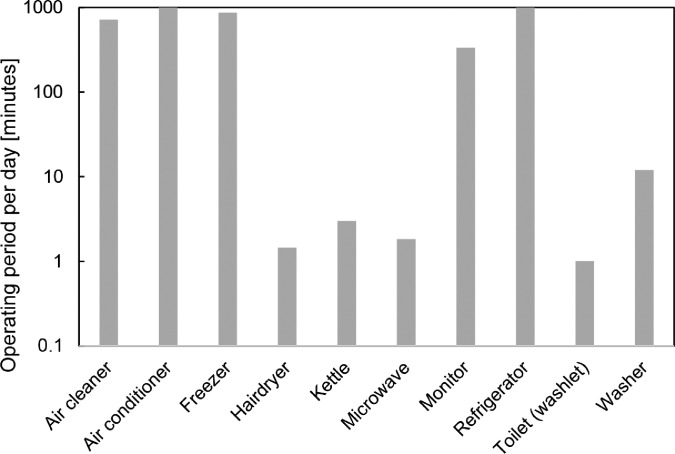
Average usage period per day based on the electric power data by MZK-EX300NM.

These data represent the electric power, voltage, and ampere of electric appliances. These data make determining when each appliance was used possible. The average operating period of each electric appliance per day is shown in Fig. 3. The air cleaner, air conditioner, and refrigerator were almost always operating; therefore, the period is about 1000 min. On the contrary, the usage of the hairdryer, kettle, microwave, and shower equipment in the bathroom is just a few minutes per day.

##### 1.1.3.2 Electric Power Data by Power Consumption Monitor

**Table:** 

Column name	Column ID	Type	Explanation
ID	id	Int	ID of each datum
Sensor name	name	String	TEPCO
Date	date	DATETIME	Measurement time
Room ID	room	Int	Room ID where the experiment took place
Total power consumption	kWh	Float	Total power consumption of the room (kWh)
Time stamp	time stamp	DATETIME	Time stored in database

**Fig. 4:**
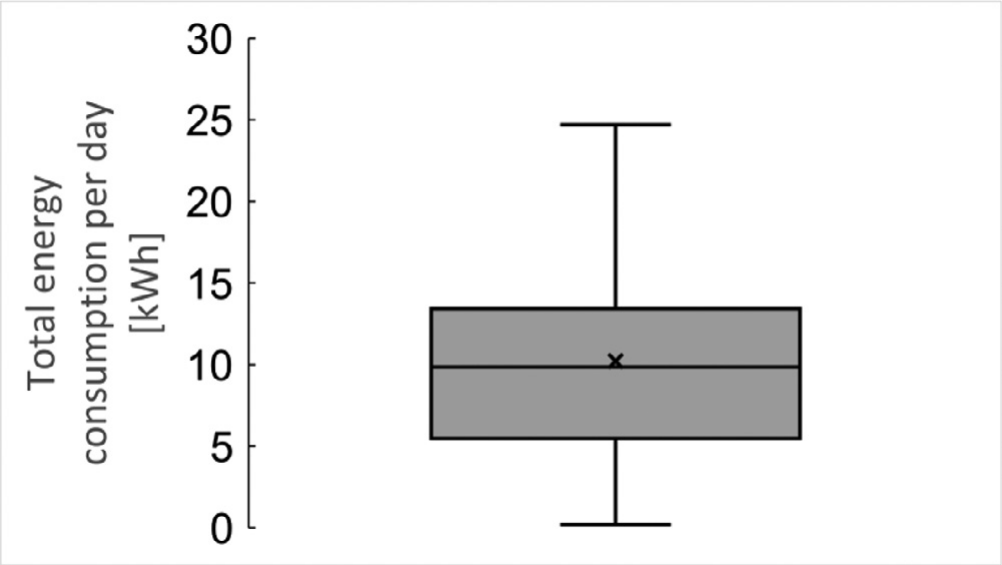
Total energy consumption per day based on the data by Power Consumption Monitor.

These data represent the total power consumption of each room. Fig. 4 represents the boxplot of average total power consumption per day.

#### 1.1.4 Activity Game Log Data

**Table:** 

Column name	Column ID	Type	Explanation
ID	id	Int	ID of each datum
Sensor name	name	String	ringfit
Date	date	DATETIME	Measurement time
Room ID	room	Int	Room ID where the experiment took place
Energy expenditure	kcal	FLOAT	Calories burned during play
Date of the play	playdate	String	Date of the play
Time of the play	playtime	INT	Play time (seconds)
Screenshot URL	screenshot_url	String	Nonpublic data
Distance run	mileage	FLOAT	Distance traveled by running exercise
Time stamp	time stamp	DATETIME	Time stored in database

**Fig. 5:**
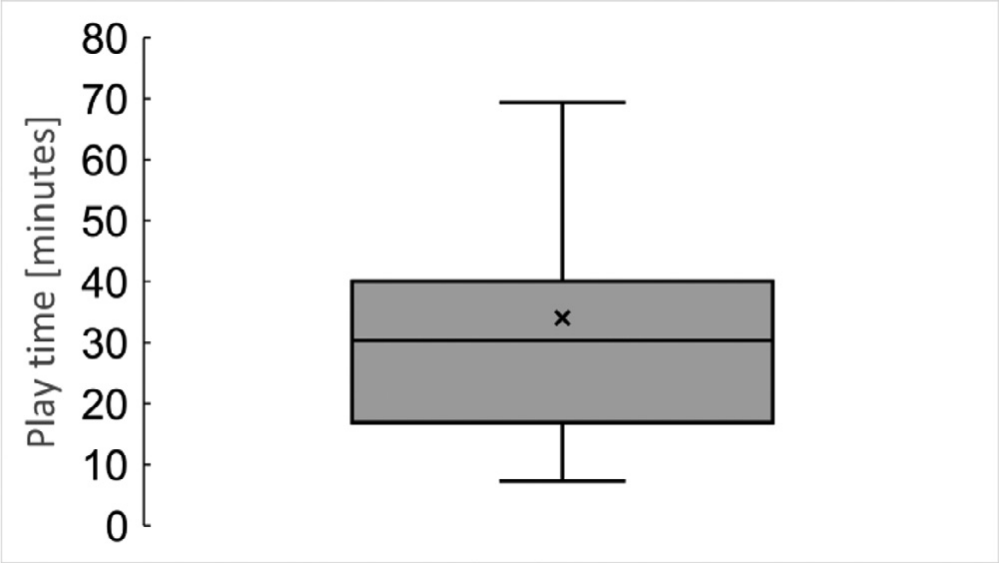
Play time per day based on the activity game log data.

These data represent when each subject plays a Nintendo Switch per day. Several subjects did not use it. Fig. 5 shows the results of creating a boxplot diagram with the data extracted only from days played for more than one minute.

#### 1.1.5 Body Composition Data

**Table:** 

Column name	Column ID	Type	Explanation
ID	id	Int	ID of each datum
Sensor name	name	String	Aria
Date	date	DATETIME	Measurement time
Room ID	room	Int	Room ID where the experiment took place
Body weight	weight	Float	Body weight value (0.01 kg unit)
BMI	bmi	Float	Calculated BMI
Body fat	fat	Float	Body fat value (0.01% unit)
Log ID	logId	String	Log ID (unique in each person)
Time stamp	time stamp	DATETIME	Time stored in database

**Fig. 6:**
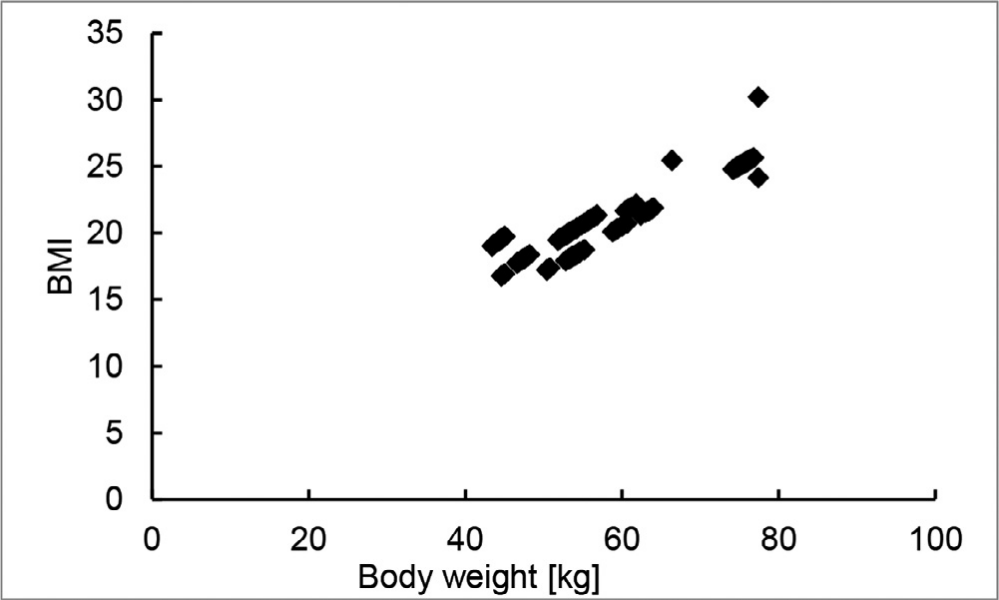
The relationship between body weight and BMI based on body composition data.

These data represent the body weight, BMI, and body fat of each subject. The BMI was calculated by body height and the body weight. The relationship between body weight and BMI is shown in Fig. 6. Body weight varies from 40 to 80 kg. Weight and BMI are roughly correlated but not perfectly because the height of each subject is different.

#### 1.1.6 Activity Level Data

**Table:** 

Column name	Column ID	Type	Explanation
ID	id	Int	ID of each datum
Sensor name	name	String	Fitbit
Date	date	DATETIME	Measurement time
Energy expenditure	calories	Float	Energy expenditure per hour (kcal)
Steps	steps	Int	Number of steps taken per hour
Distance	distance	Float	Distance traveled per hour, in meters
Floors	floors	Int	Number of floors climbed per hour
Elevation	elevation	Float	Elevation gain per hour
Room ID	room	Int	Room ID where the experiment took place
Time stamp	time stamp	DATETIME	Time stored in database

**Fig. 7:**
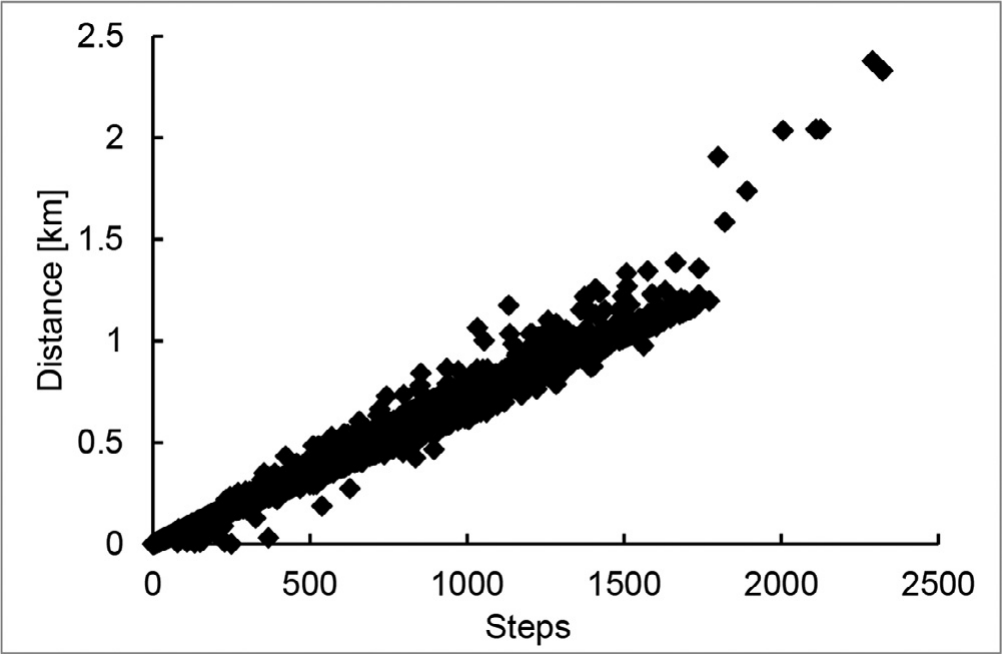
The relationship between steps and distance based on the activity level data.

These data represent the activity level of each subject, such as the number of steps, the distance traveled, and the number of floors climbed. Fig. 7 shows the relationship between the steps and distance. Because the height of each subject is different, it is not a perfect correlation.

#### 1.1.7 Sleep Data

##### 1.1.7.1 SLEEP Data by Fitbit Charge3

**Table:** 

Column name	Column ID	Type	Explanation
ID	id	Int	ID of each datum
Sensor name	name	String	Fitbit
Date	date	DATETIME	Measurement time
Room ID	room	Int	Room ID where the experiment took place
Sleep level	level	String	Awake, light, REM, deep
Seconds	seconds	Int	Number of seconds at the relevant sleep level; reset each time the sleep level changes
Time stamp	time stamp	DATETIME	Time stored in database

**Fig. 8:**
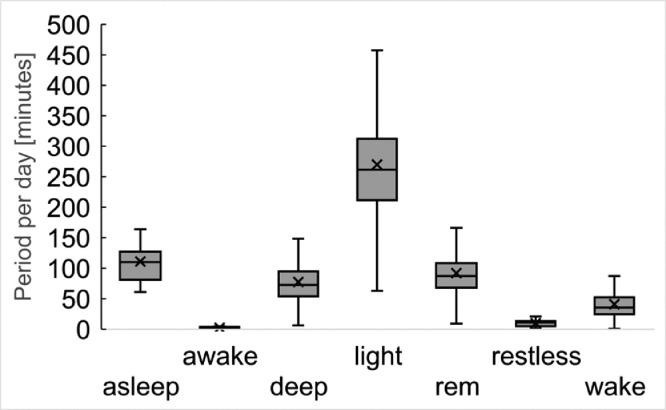
Period of each sleep state per day based on the sleep data by Fitbit Charge3.

These data represent the sleep level and the period for each subject, which was collected using Fitbit Charge3. Sleep levels are defined as asleep, awake, deep, light, rem, restless, and wake. A detailed explanation of each level is available on the Fitbit's developer site.^1^ Fig. 8. shows the boxplot of each period for each sleep level per day.

##### 1.1.7.2 Sleep Data by Withings Sleep (Sleep Level)

**Table:** 

Column name	Column ID	Type	Explanation
ID	id	Int	ID of each datum
Sensor name	name	String	Withings
Date	date	DATETIME	Measurement time
Room ID	room	Int	Room ID where the experiment took place
Start time	startdate	DATETIME	The start time of the sleep level
End time	enddate	DATETIME	The end time of the sleep level
Sleep level (ID)	state	Int	0,1,2,3
Sleep level (Text)	state_str	String	awake, light, deep, rem
Time stamp	time stamp	DATETIME	Time stored in database

**Fig. 9:**
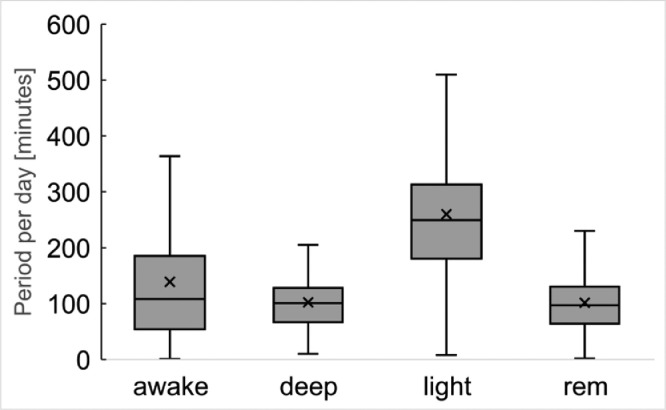
Period of each sleep state per day based on the sleep data by Withings Sleep (sleep level).

These data represent sleep levels and the start and end times for each subject, which was collected using Withings Sleep. Fig. 9 represents the boxplot of the period of each sleep level per day. Because the Fitbit Charge3 is a smartwatch and the Withings Sleep is a device in the bed, there are some differences between the sleep data compiled by the two.

##### 1.1.7.3 Sleep Data by Withings Sleep (Sleep Event)

**Table:** 

Column name	Column ID	Type	Explanation
ID	id	Int	ID of each datum
Sensor name	name	String	Withings
Date	date	DATETIME	Measurement time
Room ID	room	Int	Room ID where the experiment took place
Event name	data_field	String	Heart_rate, Respiration_rate, or Snoring_time
Value	value	Int	Value for each event
Time stamp	time stamp	DATETIME	Time stored in database

**Fig. 10:**
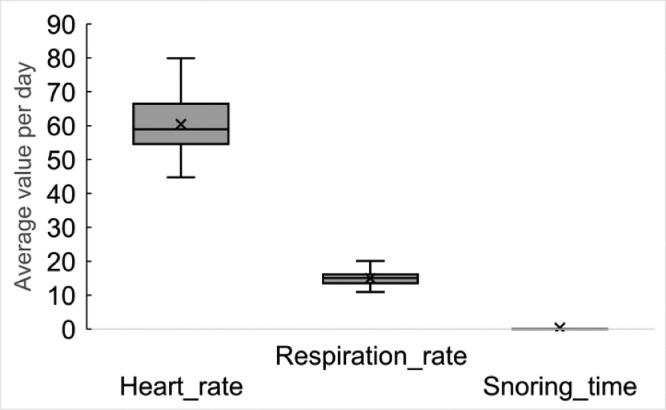
Average value of each sleep event per day sleep data by Withings Sleep (sleep event).

These data represent the heart rate, respiration time, and snoring time. Fig. 10 shows the average value per day. Because of the relatively young age of the subjects, snoring was barely detectable.

#### 1.1.8 Heart Rate Data

**Table:** 

Column name	Column ID	Type	Explanation
ID	id	Int	ID of each datum
Sensor name	name	String	Fitbit
Date	date	DATETIME	Measurement time
Room ID	room	Int	Room ID where the experiment took place
Heart rate	value	Int	Heart rate value
Time stamp	time stamp	DATETIME	Time stored in database

**Fig. 11:**
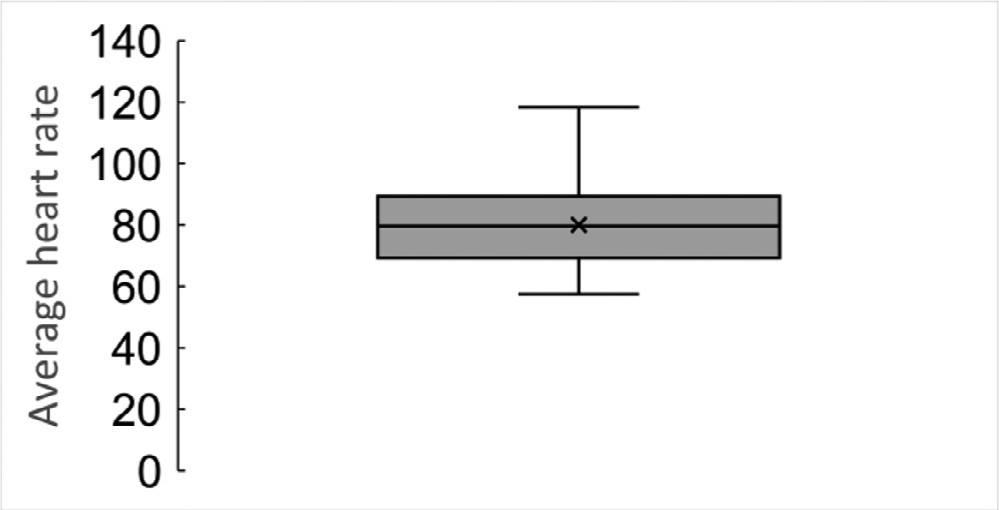
Average heart rate per day based on the heart rate data.

These data represent the heart rate of each subject every minute. The average heart rate per day is shown in Fig. 11.

#### 1.1.9 Environmental Data

##### 1.1.9.1 Environmental Data by Nature Remo

**Table:** 

Column name	Column ID	Type	Explanation
ID	id	Int	ID of each datum
Sensor name	name	String	Nature Remo
Date	date	DATETIME	Measurement time
Room ID	room	Int	Room ID where the experiment took place
Place	place	String	Location
Humidity: value	hu_val	Float	Humidity value (1% unit)
Humidity: time	hu_created_at	DATETIME	Update time of humidity value
Illuminance: value	il_val	Float	Illuminance (from 0 to 255)
Illuminance: time	il_created_at	DATETIME	Update time of illuminance value
Human perception: value	mo_val	Float	From 1 to 4
Human perception: time	mo_created_at	DATETIME	Update time of human perception value
Temperature: value	te_val	Float	Temperature value (0.1° unit)
Temperature: time	te_created_at	DATETIME	Update time of temperature value
Time stamp	time stamp	DATETIME	Time stored in database

**Fig. 12:**
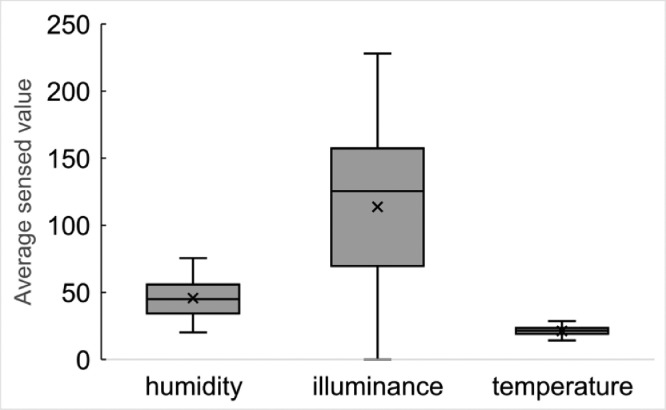
Average sensed values per day based on the environmental data by Nature Remo.

These data represent humidity, illuminance, human perception, and temperature every minute. Fig. 12 shows the boxplot of the average sensed values per day.

##### 1.1.9.2 Environmental Data by WS-USB01-THP

**Table:** 

Column name	Column ID	Type	Explanation
ID	id	Int	ID of each datum
Sensor name	name	String	THP
Date	date	DATETIME	Measurement time
Room ID	room	Int	Room ID where the experiment took place
Temperature	temperature	Float	Temperature value (0.01° unit)
Humidity	humidity	Float	Humidity value (0.01% unit)
Atmospheric pressure	atmosphere	Float	Atmospheric pressure (0.01hPa unit)
Place	place	String	Location
Time stamp	time stamp	DATETIME	Time stored in database

**Fig. 13:**
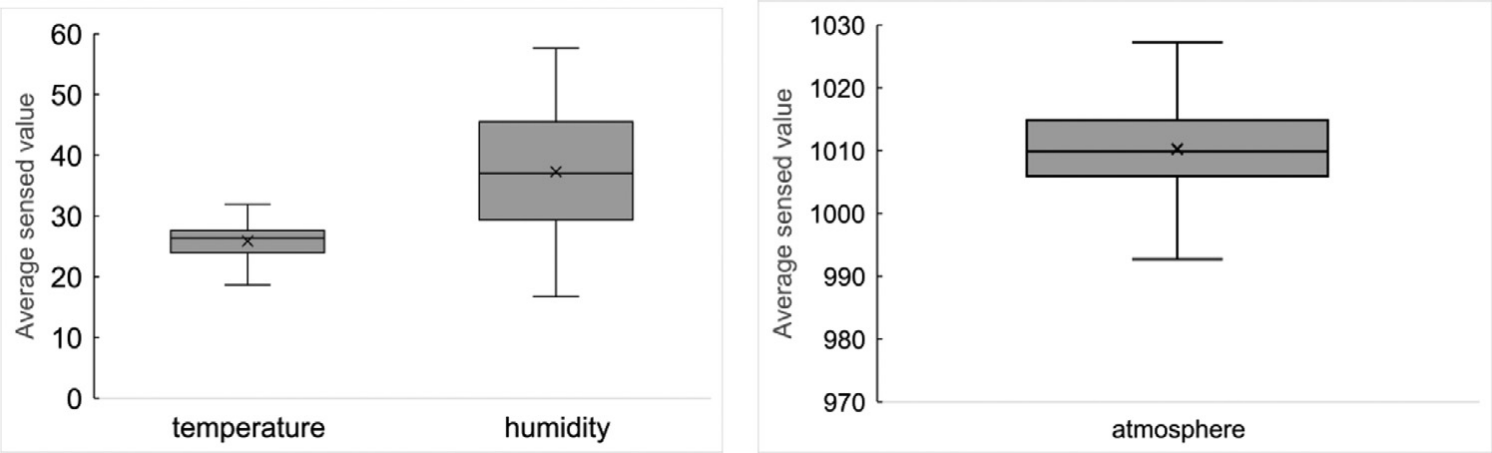
Average sensed values per day based on the environmental data by WS-USB01-THP.

These data represent humidity, temperature, and atmosphere in every minute. Fig. 13 shows the boxplot of the average sensed values per day based on these data.

## 2 Experimental Design, Materials and Methods

### 2.1 Subjects

The dataset contains the data of 23 subjects (16 male and 7 female). They were young people between the ages of 20 and 38. Most of them lived in Tokyo. The Ethics Committee of the University of Electro-Communications in accordance with the Declaration of Helsinki approved this experiment (Management ID: 19,066), and written consent was obtained from the subject. Data from subjects who consented to data release but were found [1] to have potentially high rates of individual re-identification were excluded.

They were asked to wear a smartwatch and live a normal life in the smart room. During the day, they were free to go to college or work. However, due to the effects of COVID-19, some subjects spent most of the day in the room.

To ensure that the sensors were working properly, a video camera was installed in the room. However, the subjects were free to turn off the camera at will.

### 2.2 Experimental Smart Rooms

**Fig. 14:**
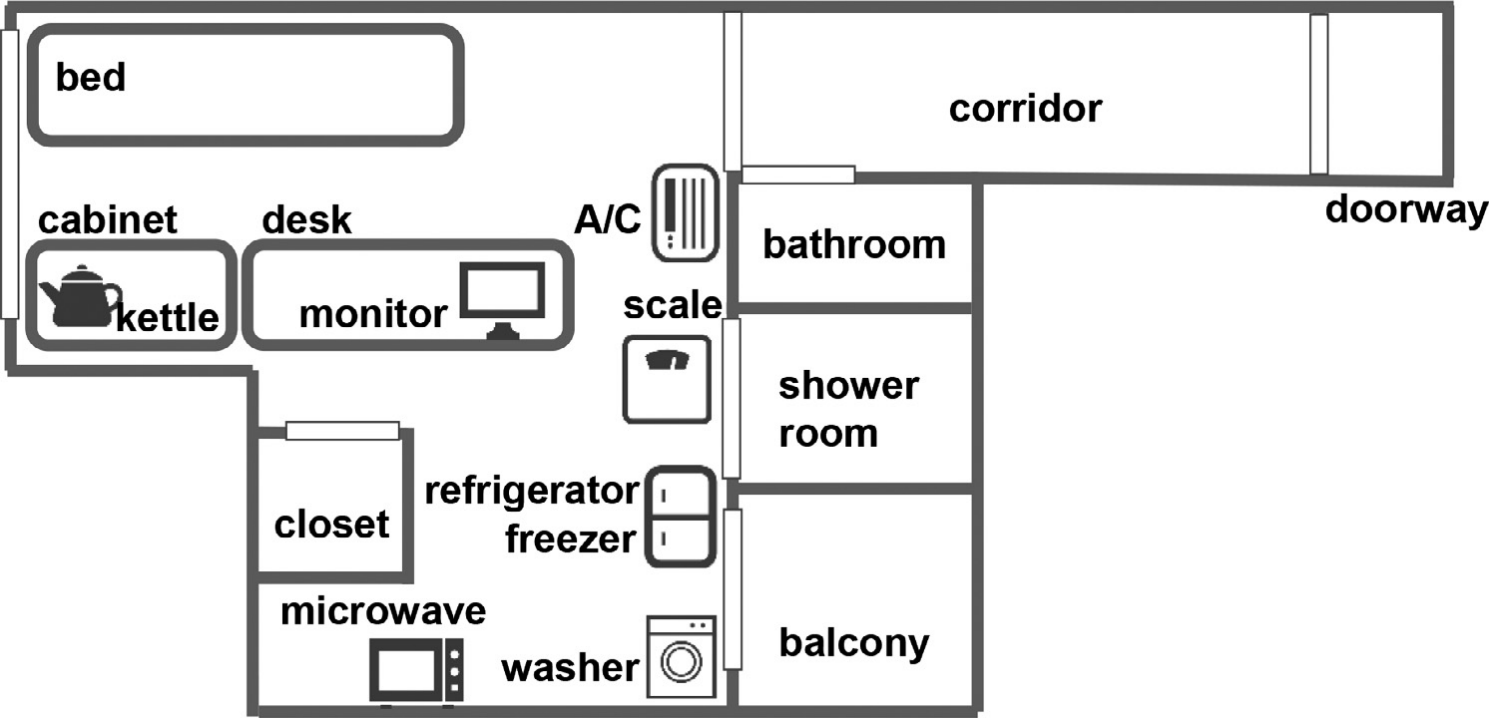
Overview of smart room 1.

**Fig. 15:**
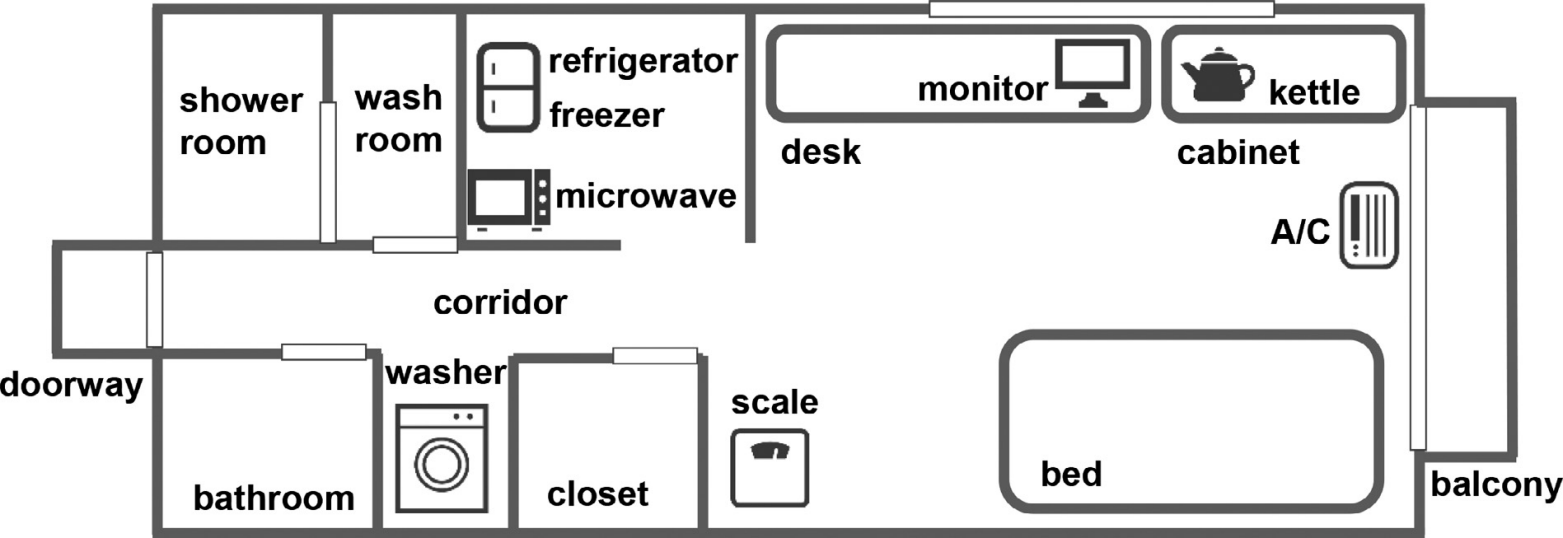
Overview of smart room 2.

Two smart rooms were prepared in Tokyo, Japan. The room size, monthly rent, and facilities were standard for Tokyo [2,3]. The overviews of the two smart rooms are shown in Figs. 14 and 15, respectively.

### 2.3 Data Collection System

**Fig. 16:**
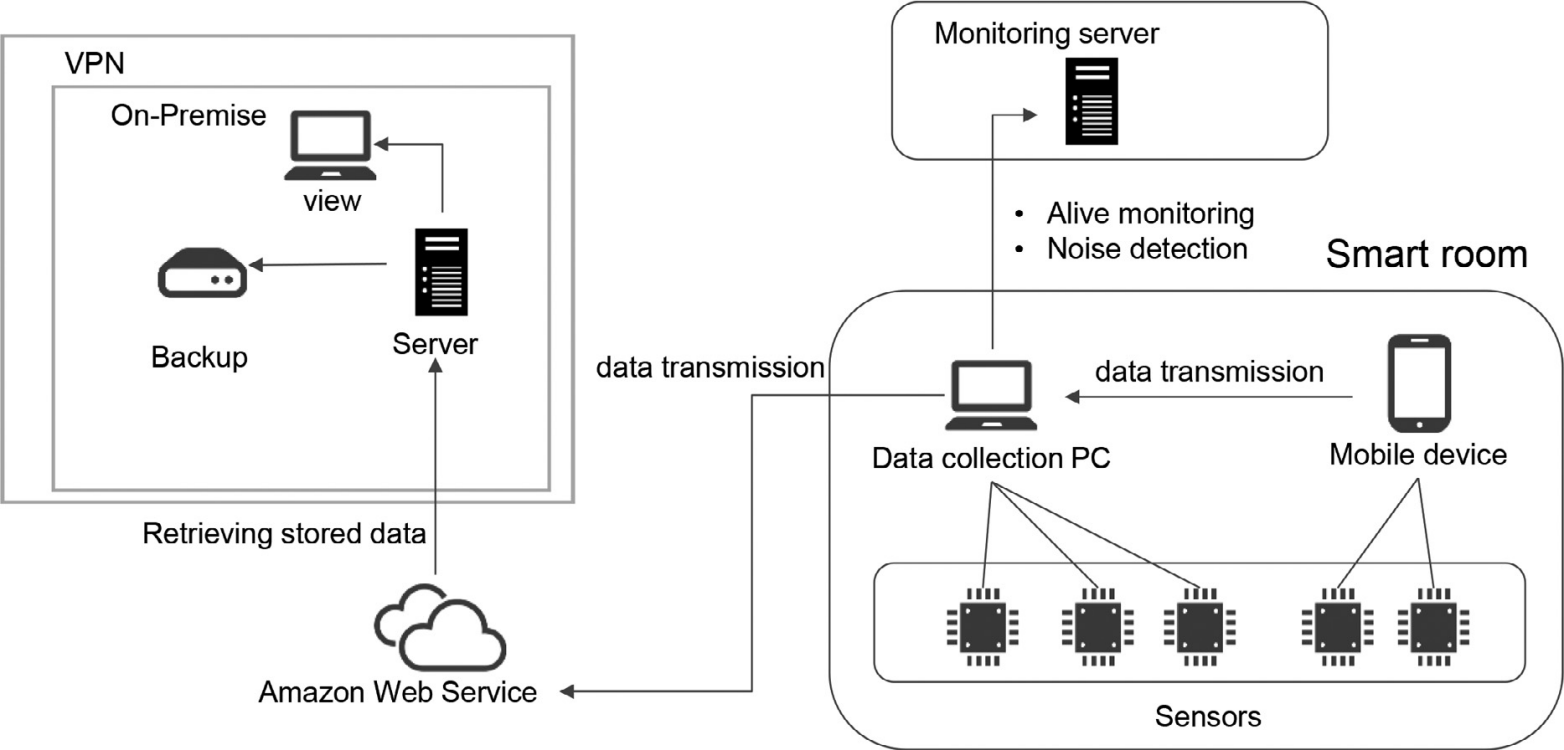
Data collection system.

We developed a data collection system, which controlled all sensing systems and stored the collected data in Amazon Simple Storage Service (Amazon S3), which is part of Amazon Web Services (AWS). Fig. 16 shows the overview of the data collection system. Data to be linked to the apps were routed through the mobile device once. The collected data can be used for the analysis of the sleep state and the daily activities [4], [5], [6], for example.

#### 2.3.1 Behavioral Data

##### Open/Closed Sensor Data

**Fig. 17:**
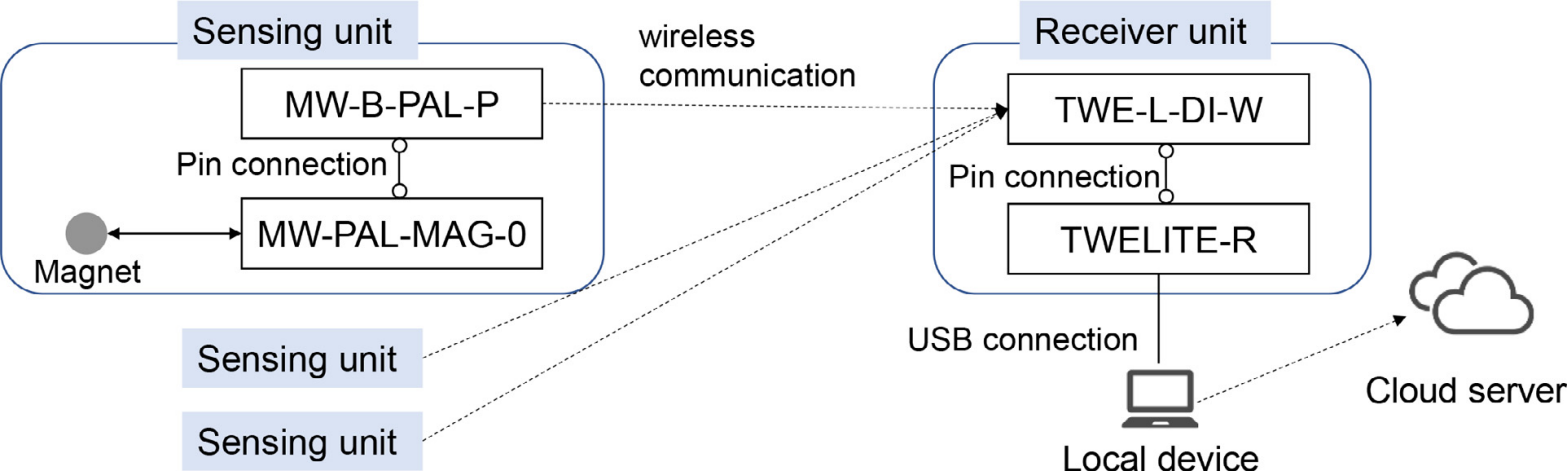
Overview of open/closed sensing system.

MW-PAL-MAG-0 was used as a magnet sensor. By attaching a magnet to the door frame and the MW-PAL-MAG-0 to the door, the MW-PAL-MAG-0 can detect the magnet when the door is closed. This allows the state of the door's opening and closing to be known. Fig. 17 shows the open/closed sensing system.

For example, the sensors were installed on microwave oven doors, washing machine lids, toilet seat lids, and entrance doors. Below is a list of the locations where these sensors were installed:

• Wind outlet of the air conditioner
• Door of the balcony
• Door of the bathroom
• Door of the closet
• Door connecting the corridor and the room (room 1 only)
• Frame of the doorway
• Door of the shower room
• Door of the washroom (room 2 only)
• Door of the microwave
• Door of the refrigerator
• Door of the freezer
• Door of the washer
• Toilet lever
• Toilet seat

##### Human Detection Sensor Data

WS-USB02-PIR was used to detect humans.

##### Electric Power Data

MZK-EX300NM and Power Consumption Monitor were used. MZK-EX300NM was installed to measure the electric power, voltage, and ampere of several consumer electronics. Below is a list of the consumer electronics:

• Kettle
• Toilet
• Hairdryer
• Monitor
• Air cleaner
• Washer
• Microwave
• Freezer
• Air conditioner
• Refrigerator

Power Consumption Monitor measured the total power consumption of each room.

##### Activity Game Log Data

Nintendo Switch is one of the most commonly played game consoles in homes around the world. Joy-Con is a controller for Nintendo Switch. Joy-Con is equipped with an accelerometer and gyro-sensor; one Joy-Con is stored in Ring-Con, and the other Joy-Con is stored in the leg strap. The user holds the Ring-Con in his or her hand and wraps the leg strap around the thigh to enjoy the game. Joy-Con measures the calories burned during play, playtime duration, and distance traveled during running exercises.

#### 2.3.2 Biometric Data

##### Body Composition Data

Fitbit Aria 2 was used. The data of body weight, BMI, and body fat were measured.

##### Activity Level Data

Fitbit Charge3 was used. Energy expenditure per hour, number of steps taken per hour, distance traveled per hour, number of floors climbed per hour, and elevation gain per hour were measured.

##### Sleep Data

Fitbit Charge3 and Withings Sleep were used. Fitbit Charge3 can measure the sleep level (awake, light, REM, and sleep), and Withings Sleep can measure the sleep level (awake, light, deep, and REM) and sleep events (heart rate, respiration rate, and snoring time.)

##### Heart Rate Data

Fitbit Charge3 was used. This watch is waterproof, but some subjects remove it when showering. The heart rate during sleep is also included in the sleep data measured by Withings Sleep.

#### 2.3.3 Environmental Data

We used two sensors: Nature Remo and WS-USB01-THP. The data collected by Nature Remo include Humidity, illuminance, temperature, and human perception. The data collected by WS-USB01-THP include humidity, atmosphere, and temperature.

## 3 Discussion

### 3.1 Benefit

The proposed dataset can benefit many types of professionals: researchers of privacy-preserving data mining, engineers of a simulator of human activities in a household, engineers of healthcare applications, people studying machine learning algorithms, etc. The details are as follows.

*Researchers of privacy-preserving data mining:* These researchers require datasets containing people's attribute values, behaviors, and so on. Many datasets contain only attribute values, such as age and gender; however, to our knowledge, no large datasets include personal attribute values, health status, and daily behavioral data. Researchers can benefit from the proposed dataset because each algorithm can be evaluated based on real data, which includes various personal values.

*Engineers of a simulator of human activities in a household:* In recent years, efforts to construct digital twins have expanded to model people's behavior. To simulate people's behavior in a household, simulator engineers need to understand what kind of person does what activity. Since the proposed dataset contains time-series data of people's daily lives, engineers can utilize the proposed dataset to build simulators for digital twins, etc.

*Engineers of healthcare applications:* Daily activities may affect the quality of sleep at night, but the specific relationship is not fully clear. Analysis of the proposed dataset may afford behavioral recommendations that lead to better sleep. Analyzing the impact of the indoor environment (brightness, temperature, and humidity) on sleep quality would also be possible.

*People studying machine learning algorithms:* By utilizing machine learning algorithms, a variety of predictive tasks can be performed. For example, an algorithm can be implemented to improve the accuracy of predicting which action to take within the next hour (take a shower, go to the bathroom, etc.). Predicting the depth of sleep based on changes in the heart rate during sleep would also be possible. Since the proposed dataset contains a variety of data, it is useful for a wide range of learners, including simple tasks and prediction of time-series data. Furthermore, for study purposes, using a dataset that can be of interest to the learner is preferred. Behavioral data from daily life is likely to be of interest.

### 3.2 Usage Examples of the Dataset

The dataset was used in [1] to evaluate the Laplace mechanism, the most famous algorithm for protecting personal privacy. The Laplace mechanism is an algorithm that realizes differential privacy, a notion for publicly sharing data with compromising personal privacy. Differential privacy has been used in many organizations, such as Google, Apple, and Microsoft. This dataset was performed on the Laplace mechanism, and the authors of [1] showed that each individual's privacy can be protected as long as the individual's data is not an outlier.

Machine learning models that predict people's behaviors can be generated based on the proposed dataset. By publishing such machine learning models, more researchers and practitioners can benefit from the proposed dataset. Moreover, visualization of the proposed dataset would increase its usefulness. For example, an individual's sequence of actions can be represented in a flowchart diagram. Such processing is a subject for future research.

## Ethics Statements

The Ethics Committee of the University of Electro-Communications in accordance with the Declaration of Helsinki approved this experiment (M0061nagement ID: 19,066), and written consent was obtained from the subject.

## CRediT authorship contribution statement

**Yuichi Sei:** Conceptualization, Methodology, Software, Validation, Formal analysis, Investigation, Resources, Data curation, Writing – original draft, Writing – review & editing, Visualization, Project administration, Funding acquisition. **Akihiko Ohsuga:** Conceptualization, Supervision.

## Declaration of Competing Interest

The authors declare that they have no known competing financial interests or personal relationships that could have appeared to influence the work reported in this paper.

## Data Availability

Biomedical data and sensing information in smart rooms (Original data) (Mendeley Data).

## Footnotes

1 https://dev.fitbit.com/build/reference/web-api/sleep/
